# THE TELLEGACY PROGRAM

**DOI:** 10.1093/geroni/igac059.2161

**Published:** 2022-12-20

**Authors:** Jeremy Holloway

**Affiliations:** University of North Dakota, Fargo, North Dakota, United States

## Abstract

Emerging research displays how social isolation and loneliness (SIAL) before and during the Coronavirus pandemic are linked to health issues caused by SIAL. These issues are estimated to increase the risk of early death by 26 percent, the equivalency of smoking 15 cigarettes a day. The program started soon after the pandemic as an intergenerational program created to combat SIAL among older adults. University students, known as Legacy Builders, connect through virtual or via phone conversations to communicate meaningfully with the older adult. Students are prepared with a curriculum on mindfulness, SMART goal-setting, growth mindset, visualization goal-setting exercises, and reminiscence therapy as well as the skill and art of listening. At the end of the encounters, a legacy book is given to the older adult with a description of the older adult’s life shared during their conversations with the Legacy Builder. The UCLA Loneliness Scale was utilized along with older adult self-reports. Sixteen residents in a memory care facility, ages 58 to 86, with multiple levels of dementia participated in the program of Summer 2021. The UCLA Loneliness Scale revealed that 13 of the 16 residents originally reported that they feel lonely or isolated. After 5 weeks of participation 7 out of the 16 residents report that they felt less lonely. Qualitative analysis showed improved perceptions of aging and an interest in Geriatrics among the student participants. These results also displayed positive older adult perceptions of the younger generation, and similar responses of the younger generation towards older adults.

